# Surgical Strategy for Squamous Cell Carcinoma of the External Auditory Canal: Management of Locally Advanced Cases with Skull Base Involvement

**DOI:** 10.1055/a-1733-2585

**Published:** 2022-02-04

**Authors:** Seiya Goto, Naoki Nishio, Kenichiro Iwami, Tadao Yoshida, Takashi Maruo, Nobuaki Mukoyama, Hidenori Tsuzuki, Sayaka Yokoi, Akihisa Wada, Mariko Hiramatsu, Yuichiro Hayashi, Yuzuru Kamei, Masazumi Fujii, Michihiko Sone, Yasushi Fujimoto

**Affiliations:** 1 Department of Otorhinolaryngology, Nagoya University Graduate School of Medicine, Nagoya, Japan; 2 Department of Neurosurgery, Aichi Medical University, Aichi, Japan; 3 Graduate School of Informatics, Nagoya University, Nagoya, Japan; 4 Department of Plastic and Reconstructive Surgery, Nagoya University Graduate School of Medicine, Nagoya, Japan; 5 Department of Neurosurgery, Fukushima Medical University, Fukushima, Japan; 6 Department of Otorhinolaryngology, Head and Neck Surgery, Aichi Medical University, Aichi, Japan

**Keywords:** external auditory canal cancer, squamous cell carcinoma, surgical strategy, overall survival, disease-specific survival, T4 subclassification

## Abstract

**Objective** Surgical indications for advanced-stage squamous cell carcinoma (SCC) of the external auditory canal (EAC) are highly dependent on the skull base surgery team. The aim of this study was to evaluate the surgical outcomes in patients with SCC of the EAC and to clarify the surgical indication of far advanced cases using the T4 subclassification.

**Methods** Patients with SCC of the EAC who underwent curative treatment from 2002 to 2021 at our hospital were retrospectively reviewed. Clinical and surgical results, including operative data, overall survival (OS), and disease-specific survival (DSS), were analyzed. To clarify the surgical indication for advanced-stage tumors, we proposed the T4 subclassification.

**Results** In the 46 patients included in the study, 8 patients had T1 tumors, 10 had T2 tumor, 5 had T3 tumors, and 23 had T4 tumors. The 5-year DSS with T1, T2, T3, and T4 tumors were 100, 85.7, 100, and 61.7%, respectively. No prognostic impacts for margin status were found between the 5-year OS and DSS ( *p* = 0.23 and 0.13, respectively). Patients with far-advanced-stage (T4b) tumors were significantly associated with shorter DSS than those with early-stage (T1/T2) and advanced-stage (T3/T4a) tumors ( *p* = 0.007 and 0.03, respectively).

**Conclusion** The present study focused on patients with SCC of the EAC at a university hospital over a period of 20 years, especially with skull base involvement, and a T4 subclassification was proposed. Complete tumor resection in an en bloc fashion could help achieve a good survival rate even in patients with locally advanced tumors.

## Introduction

Squamous cell carcinoma (SCC) of the external auditory canal (EAC) arises from the external ear and is a quite rare form of head and neck cancer, comprising less than 0.2% of all head and neck tumors. 1 2 Currently, the modified Pittsburgh classification, which is based on the intraoperative findings and the histopathological assessments, is a commonly used tumor classification for SCC of the EAC. 2 Complete tumor resection with free margins achieved better survival outcomes in patients with early-stage (T1/T2) tumors. 3 However, because of the unique regional anatomy, only few symptoms are experienced in its early stage, such as otorrhea, itching, and swelling of the ear canal, resulting in development of a locally advanced-stage (T3/T4) tumor from the primary tumor. 4 5 6 In patients with advanced-stage (T3/T4) tumor, complete tumor resection with free margins is the mainstay of treatment, but is often challenging due to the complicated bony anatomy of the temporal bone and the surrounding crucial organs, including the internal carotid artery, cranial nerves, and brain. A meta-analysis of 274 patients with SCC of the EAC showed that the 5-year overall survival (OS) rates were 72.5 and 35.8% for T3 and T4 tumors, respectively. 7 In particular, the 5-year OS rate for T4 tumors was 29.5 to 46.3%, with a poor prognosis. 8 9 10

Surgery is the mainstay of treatment for patients with SCC of the EAC, and most clinicians agree that, if possible, complete tumor resection in an en bloc fashion should be performed for both early-stage (T1/T2) and advanced-stage (T3/T4) tumors. Craniofacial resection enables us to perform complete en bloc resection of the tumor with tumor-free margins, even for locally advanced skull base malignancies, 11 and thus, could be the first choice of treatment even in patients with locally advanced EAC cancer. 12 Recent studies demonstrated that surgical resection achieved better survival results with a low risk of severe complication in both early 3 and advanced EAC cancers. 12 13 However, craniofacial resection in temporal bone malignancies requires experienced surgical skills and sufficient anatomical knowledge of the skull base regions. Therefore, surgical indications for advanced-stage SCC of the EAC are highly dependent on the skull base surgery team at each hospital, and it remains challenging to manage the far-advanced cases involving the cochlea, medial wall of the middle ear, or the petrosal apex. Current staging systems, such as the modified Pittsburgh classification, classify the advanced tumors as only T4 tumors and do not discriminate tumors as resectable (in en bloc fashion) or unresectable. 14 In this study, we reviewed our surgical experience in the management of SCC of the EAC in a tertiary hospital with a consistent surgical strategy. The aim of this study was to evaluate the surgical outcomes in patients with SCC of the EAC using several surgical techniques and to clarify the surgical indications of far-advanced cases using the T4 subclassification.

## Materials and Methods

### Patients

The clinical charts of patients with SCC of the EAC who underwent curative treatment between 2002 and 2021 at Nagoya University Hospital were retrospectively reviewed. Patients with a different pattern on histological examination; with metastases of the carcinoma to distant areas such as the bone, lungs, and liver; or who refused to receive curative treatment were excluded from the study. Clinical and surgical results, including operative data, overall survival, and disease-specific survival (DSS) were analyzed. This study was approved by the ethics review committee of Nagoya University Hospital (2020–0162) and was performed in accordance with the Helsinki Declaration of 1975 and its amendments.

### Treatment Strategy

The main treatment strategy for SCC of the EAC was complete tumor resection in an en bloc fashion. All patients were preoperatively assessed using thin-slice computed tomography (CT) and other imaging modalities, including magnetic resonance imaging (MRI), ultrasound, and ^18^ F-fluorodeoxyglucose positron emission tomography/computed tomography (PET/CT). In our hospital, CT and MRI were evaluated by two radiologists, who were blinded to the clinical outcomes, and the diagnostic information, such as the invasion pattern in the surrounding tissues, was reported as a diagnostic radiological report. Based on this diagnostic report, tumor and nodal (TN) staging was determined according to the modified Pittsburgh classification. 2 15 Further, an appropriate surgical procedure was determined based on the preoperative assessment of tumor involvement in the surrounding adjacent tissues.

Surgery was performed by a multidisciplinary skull base surgery team, including the head and neck surgeons, neurosurgeons, and the plastic and reconstructive surgeons. During surgery, to perform accurate tumor resection, a Vector Vision Compact Navigation System (BrainLAB, Munich, Germany) was used to determine the positional relationship between the tumor and surrounding normal tissues. 16 Because of the difficulty in achieving an en bloc resection, patients with involvement of the petrous apex, carotid canal, or jugular foramen were not indicated for surgical resection.

It is recommended that postoperative radiotherapy (a dose of 50–60 Gy at 2 Gy per fraction and 5 fractions per week to the tumor bed) should be administered within 8 weeks of tumor resection, based on the extent of tumor invasion, margin status, and the patient's general condition. 17 Cisplatin-based chemoradiotherapy is recommended as an adjuvant treatment in patients with positive margins or extranodal spread.

### Surgical Techniques with or without Craniotomy

**Fig. 1:**
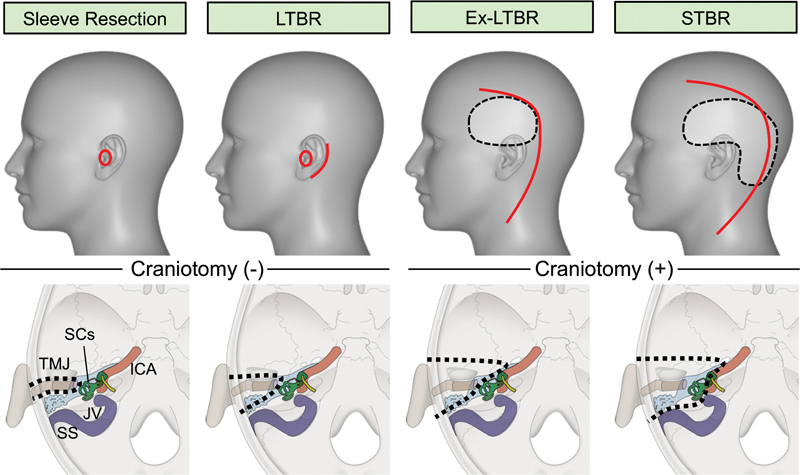
Surgical procedures for squamous cell carcinoma of the external auditory canal. **Upper:** Representative designs of skin incisions ( *red line* ) and craniotomies ( *black dotted line* ) of surgical procedures for external auditory canal cancer; sleeve resection, lateral temporal bone resection (LTBR), extended lateral temporal bone resection (Ex-LTBR), and subtotal temporal bone resection (STBR). **Lower:** Resection line of the intracranial skull base ( *black dotted line* ) and surrounding organs, including the temporomandibular joint (TMJ), internal carotid artery (ICA), sigmoid sinus (SS), jugular valve (JV), and semicircular canals (SCs).

Representative designs of skin incisions and craniotomies for each surgical classification are shown in Fig. 1 . For the early-stage (T1/T2) tumors, lateral temporal bone resection (LTBR) was generally performed, in which the tumor was resected en bloc with the surrounding normal tissue. This procedure was performed using a tympanic approach, without craniotomy. Sleeve resection was performed only in the selective patients with early-stage T1 tumors. For the locally advanced-stage (T3/T4) tumors, an extended-LTBR (Ex-LTBR) or subtotal temporal bone resection (STBR) was performed. Head and neck surgeons and neurosurgeons performed Ex-LTBR and STBR using intracranial techniques, and the surgical defects of the cranial base were covered using a free flap from the omentum or anterolateral thigh, sometimes with a fascia for closure of the dura by plastic and reconstructive surgeons. Craniofacial resection was not performed in patients with advanced-stage tumors, whose general condition was not suitable for surgery (i.e., worse than performance status of 2, as defined by the Eastern Cooperative Oncology Group). 18

Complications were considered if they occurred within 30 days postoperatively and were categorized into three groups, as previously reported: central nervous system (CNS) complications, wound complications, and systemic complications. 11 Cerebrospinal fluid (CSF) leak was classified as a major complication, if it lasted for more than 1 week or required operative intervention. The loss of all or part of the flap used in reconstruction and the need for surgical retreatment were considered the major wound complications.

### Statistical Analysis

OS and DSS were estimated using the Kaplan–Meier analysis, and the differences in survival rates were estimated using the log-rank test for all patients and for those requiring surgery. OS was calculated from the first visit of the patient to the day of death, or to the date of last follow-up. DSS was calculated from the first day of treatment to the day of death from the disease, or the date of last follow-up. Statistical analysis was performed using the EZR software package (version 1.35; Saitama Medical Center, Jichi Medical University, Saitama, Japan), which is a graphical user interface for R (The R Foundation for Statistical Computing, Vienna, Austria). Statistical significance was set at *p* < 0.05.

## Results

### Patient Characteristics

A total of 46 patients (24 men, 22 women; median age at the first visit: 67 years) who underwent curative treatment were investigated ( Table 1 ). The median duration of follow-up was 28 months (range: 2–224 months). Based on the T classification, the tumors were in T1 stage in 8 patients, T2 in 10, T3 in 5, and T4 in 23 patients. Fourteen patients received platinum-based combination chemotherapy before treatment. Out of the 46 patients, 30 patients (65%) underwent surgical tumor resection in an en bloc fashion, of which 2 underwent sleeve resection, 13 LTBR, 6 Ex-LTBR, and 9 STBR as a curative treatment. Postoperative radiotherapy was administered to four patients and chemoradiotherapy to two patients. Sixteen patients (35%) underwent nonsurgical treatment, including chemoradiotherapy, radiotherapy, or particle therapy.

**Table 1:** Characteristics in all 46 patients

Characteristics	No. of patients (%)
Age (y a )	67 (34–84)
**Sex**	
Male	24 (52)
Female	22 (48)
**Side**	
Right	23 (50)
Left	23 (50)
**T stage according to modified Pittsburg classification**	
1	8 (17)
2	10 (22)
3	5 (11)
4	23 (50)
**N classification**	
Presence	8 (17)
Absence	38 (83)
**Type of treatment**	
Surgery	30 (65)
Sleeve resection	2 (4)
Lateral temporal bone resection	13 (28)
Extended lateral temporal bone resection	6 (13)
Subtotal temporal bone resection	9 (20)
Radiotherapy	6 (13)
Chemoradiotherapy	8 (17)
Particle therapy	2 (4)

a Median (range).

Out of the 16 patients who did not undergo surgery, 4 patients were those with T1 tumors and 12 patients with T4 tumors. The median age of the four patients with T1 tumors was 83 years (range: 79–84 years). They received radiotherapy as an alternative therapy. Supplemental Table S1 (available in the online version) shows the tumor invasion features in patients with T4 tumors who received surgery ( *n* = 11) and those who did not ( *n* = 12). There was a trend of tumor invasion in the anterior portion of the EAC, such as the TMJ, in patients who underwent surgery for T4 tumors. In patients with T4 tumors, who did not undergo surgery, seven patients (58%) showed tumor invasion in the jugular foramen, five patients (42%) in the sigmoid sinus, and nine patients (75%) in the dura.

### Survival Outcome

**Fig. 2:**
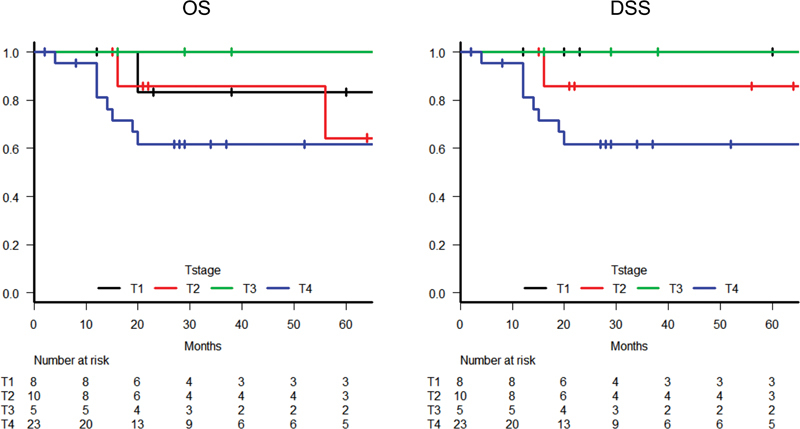
Kaplan–Meier survival curves based on the modified Pittsburgh classification for overall survival and disease-specific survival in all 46 patients with squamous cell carcinoma of the external auditory canal (DSS, disease-specific survival; OS, overall survival).

In all 46 patients, the 5-year OS and DSS rates were 69.5 and 77.6%, respectively ( Fig. 2 ). The 5-year OS rates of patients with T1, T2, T3, and T4 tumors were 83.3, 64.3, 100, and 61.7%, respectively, and the 5-year DSS rates of patients with T1, T2, T3, and T4 tumors were 100, 85.7, 100, and 61.7%, respectively.

Analysis of the 30 surgical patients showed 5-year OS and DSS rates of 85.3 and 92.4%, respectively. Among the 30 surgical patients, the 5-year OS rates of patients with T1, T2, T3, and T4 tumors were 100, 64.3, 100, and 90.0%, respectively; while the 5-year DSS rates of patients with T1, T2, T3, and T4 tumors were 100, 85.7, 100, and 90.0%, respectively ( Supplemental Fig. S1 , available in the online version). Out of the 30 patients, 26 showed negative tumor margins, while 4 showed close margins or positive margins. No prognostic impacts of margin status (negative margin vs. positive or close margins) were noticed between the 5-year OS and DSS rates ( *p* = 0.23 and 0.13).

### Operative Results in Surgical Patients

Among the 30 surgical patients, the median operation time for sleeve resection, LTBR, Ex-LTBR, and STBR were 185, 291, 720, and 881 minutes, respectively. The median amount of intraoperative blood loss during sleeve resection, LTBR, Ex-LTBR, and STBR were 38, 60, 822, and 1920 mL, respectively. The mortality rate in the 30 patients was 0%. Nine patients who received Ex-LTBR and STBR developed surgical complications after craniofacial resection, comprising a minor CSF leak in 2 patients (6.7%), a major CSF leak in 2 (6.7%), a minor wound in 5 (16.7%), and a major wound in 1 (3.3%). No other CNS complications, such as brain contusion, cerebral infarction, cerebral hemorrhage, and/or brain abscess, were observed ( Table 2 ).

**Table 2:** Mortality and complications in 30 surgical patients

Type of complications	No. of patients (%)
Mortality rate	0 (0)
Complications	9 (30)
**Central nervous system**	
Cerebral infraction	0 (0)
CSF leakage major	2 (7)
CSF leakage minor	2 (7)
Meningitis	0 (0)
Cerebral hemorrhage	0 (0)
**Wound**	
Major	1 (3)
Minor	5 (17)
Flap necrosis	0 (0)
**Systemic**	
Pneumonia	0 (0)
Sepsis	2 (7)

Abbreviation: CSF, cerebrospinal fluid.

### Tumor Recurrence in Surgical Patients

**Fig. 3:**
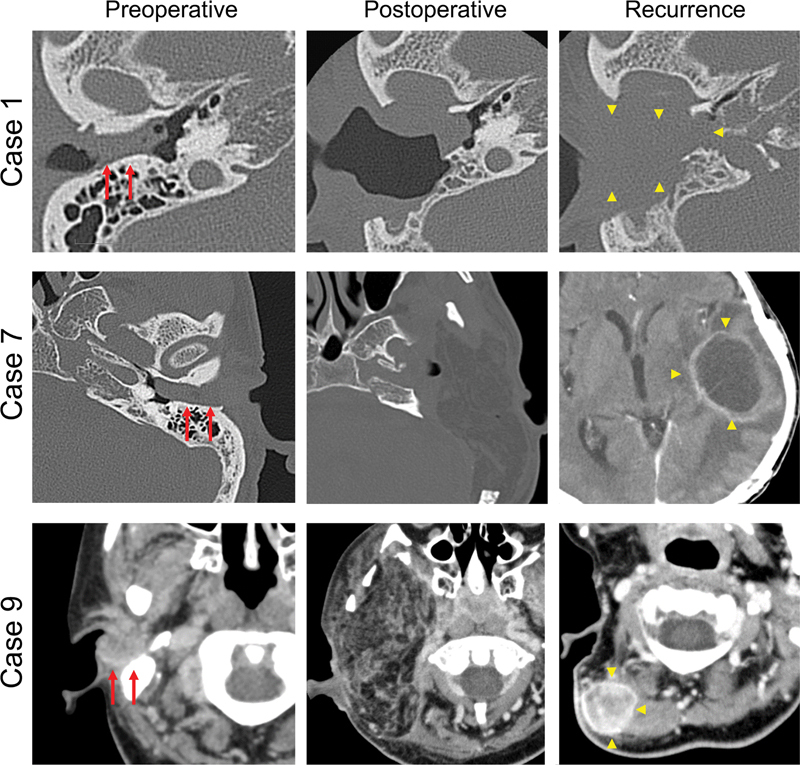
Representative computed tomography images of the three surgical patients: preoperative, postoperative, and at the time of recurrence.

Among the 30 surgical patients, 9 patients experienced tumor recurrence ( Table 3 ). Four patients experienced local recurrence after surgery, of which two died of the disease. In the three patients with early-stage tumor, two patients had local recurrences at the petrous apex and received chemoradiotherapy. One patient achieved a complete response after salvage radiotherapy, while the other died of the disease. One patient had parotid gland lymph node metastasis, but was rescued by surgical resection. Among the six patients with advanced-stage tumors, two had local recurrences, one had a posterior lymph node metastasis, and three had a distant metastasis in the lung. All patients received adjuvant chemotherapy. Representative CT images of the patients preoperatively, postoperatively, and at the time of recurrence are shown in Fig. 3 .

**Table 3:** Characteristics for patients with a recurrent tumor after surgery

Case	Age (y)	Sex	T stage	Progress	Surgery	Margin status	Follow-up (mo)	Recurrent area	Outcome
1	65	M	2	Partial bone erosion	LTBR	Negative	16	Local	DOD
2	63	M	2	Partial bone erosion	LTBR	Negative	87	Local	NED
3	66	M	3	Eroding the osseous EAC	LTBR	Close	38	Local	AWD
4	73	M	1	Limited to the EAC	LTBR	Negative	73	Regional	NED
5	57	F	4	Eroding the medial wall of middle ear	STBR	Close	72	Distant	AWD
6	60	F	4	Involvement of TMJ and dura	Ex-LTBR	Negative	27	Distant	AWD
7	54	M	4	Involvement of dura	STBR	Positive	12	Local	DOD
8	72	F	4	Extensive (>0.5 cm) soft-tissue involvement	Ex-LTBR	Negative	37	Distant	NED
9	49	F	4	Involvement of styloid process	STBR	Positive	29	Regional	AWD

Abbreviations: AWD, alive with disease; DOD, dead of disease; EAC, external auditory canal; Ex-LTBR, extended lateral temporal bone resection; F, female; LTBR, lateral temporal bone resection; M, male; NED, no evidence of disease; STBR, subtotal temporal bone resection; TMJ, temporomandibular joint.

### Surgical Indications and T4 subclassification for Locally Advanced Stage

To clarify the surgical indications for patients with locally advanced-stage tumor, an analysis was performed later. Considering the difficulty in achieving an en bloc resection and subsequent severe central complications arising in the far-advanced-stage tumors, we divided the T4 stage of tumor into T4a (resectable; tumor eroding the cochlea, medial wall of the middle ear, with extensive [> 0.5 cm] soft-tissue involvement, or evidence of facial paresis) and T4b (unresectable; tumor eroding the petrous apex, carotid canal, jugular foramen, or dura) subtypes, based on the diagnostic radiological report. According to the surgical procedures performed with or without craniotomy, EAC cancer can be classified as follows: early-stage (T1//T2), advanced-stage (T3/T4a), and far-advanced-stage (T4b) tumors.

**Fig. 4:**
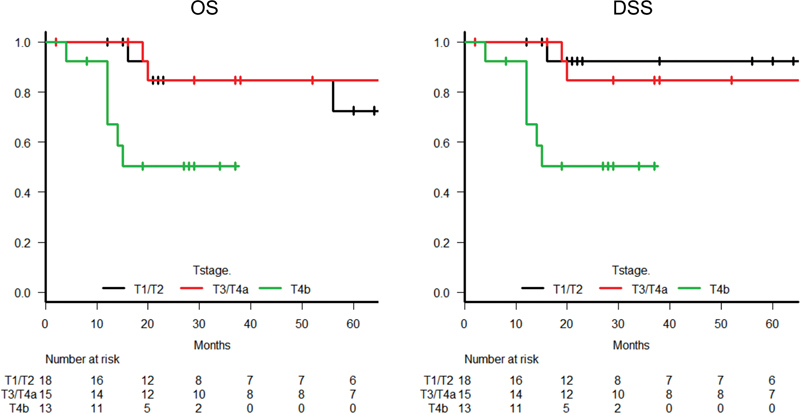
Kaplan–Meier survival curves based on the T4 subclassification of early-stage (T1/T2), advanced-stage (T3/T4a), and far advanced-stage (T4b) tumors for overall survival and disease-specific survival in all 46 patients with squamous cell carcinoma of the external auditory canal (DSS, disease-specific survival; OS, overall survival).

The survival outcomes with OS in 46 patients with early-stage (T1/T2), advanced-stage (T3/T4a), and far-advanced-stage (T4b) tumors are shown in Fig. 4 . According to our T4 subclassification, 23 patients with T4 tumor were divided into T4a (10 patients) and T4b (13 patients). Patients with a far-advanced-stage (T4b) tumor were significantly associated with shorter OS than those with early-stage (T1/T2) and advanced-stage (T3/T4a) tumors (3-year OS: 50.3 vs. 84.6 and 84.6%, *p* = 0.02 and 0.03, respectively), and shorter DSS (3-year DSS: 50.3 vs. 92.3 and 84.6%, *p* = 0.007 and 0.03, respectively).

## Discussion

**Fig. 5:**
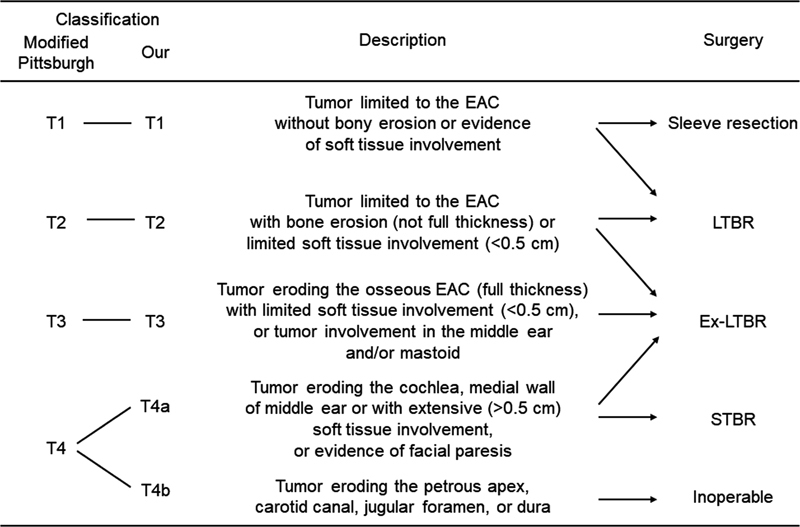
Surgical strategy for squamous cell carcinoma of the external auditory canal based on the T4 subclassification, in which T4 disease is divided into resectable cases (T4a) and unresectable cases (T4b). (EAC, external auditory canal; Ex-LTBR, extended lateral temporal bone resection; LTBR, lateral temporal bone resection; STBR, subtotal temporal bone resection.)

Surgical tumor resection with free margins is considered the most favorable treatment in patients with SCC of the EAC, 2 8 13 19 which poses the following clinical question: is the tumor in the locally advanced stage resectable or not? The cancer staging system is considered representative of tumor prognosis in most of the solid tumors. Currently, several tumor-staging classifications have been proposed for EAC cancer, including the original Pittsburgh classification, 15 the modified Pittsburgh classification, 2 the staging system proposed by Stell and McCormick, 20 or the American Joint Committee on Cancer (AJCC) staging system (8th edition). 21 Generally, the TNM classification using the AJCC 8th edition is used for head and neck cancer, in which EAC cancer is categorized in the section of ear skin cancer. However, due to the rarity of EAC cancer and the anatomical differences from other skin cancers, T-classification using the AJCC 8th edition does not reflect the clinical survival outcome. In this study, we developed a modified Pittsburgh classification to clarify surgical indications in patients with SCC of the EAC, in which the patients with T4 tumors were divided into two groups as follows: T4a (resectable) and T4b (unresectable) tumors. Using our T4 subclassification, SCC of the EAC could be classified as early-stage (T1/T2), advanced-stage (T3/T4a), and far-advanced-stage (T4b) tumors. Better survival outcomes were obtained in patients with early-stage (T1/T2) and advanced-stage (T3/T4a) tumors, with a 3-year OS of 84.6 and 84.6%, respectively, compared with 50.3% with far-advanced-stage (T4b) tumors. Our surgical strategy for patients with SCC of the EAC is shown in Fig. 5 . We initially considered the tumor invading the dura as “resectable” and removed the tumor with dural invasion in an en bloc fashion. However, the clinical outcomes in patients with dural invasion were generally poor, and the survival results were not satisfactory. Several authors have discussed the prognostic impact of dural invasion in patients with SCC of the EAC. A recent study of patients with T4 tumors in the temporal bone demonstrated that the invasion of the dura in the posterior fossa could be considered an independent prognostic factor in multivariate analysis. 22 Although we technically excised the tumor with dural invasion, we consider the tumor with dural invasion as “unresectable.” At present, the tumors with dural invasion are thought to be T4b tumors in our hospital.

In patients with far-advanced-stage (T4b) tumors, other treatment modalities such as radiotherapy or proton/heavy-ion therapy are recommended, instead of surgical resection. Concomitant chemoradiotherapy with a combination of cisplatin, 5-fluorouracil, and docetaxel can be considered an effective alternative for surgical treatment and achieved a 5-year OS rate of 56% in 34 patients with unresectable T4 tumors, 23 which might be classified as T4b tumors in our T4 subclassification. Considering the difficulty in achieving an en bloc resection, highly invasive surgery, and severe perioperative complications due to the sacrifice of the carotid artery, sigmoid sinus, or brain, we do not recommend extensive curative surgery in patients with far-advanced-stage (T4b) tumor, which invades the petrous apex, carotid canal, jugular foramen, or dura.

To achieve a safe surgical margin in EAC cancer, an appropriate surgical strategy based on preoperative imaging should be determined by a multidisciplinary skull base surgery team, including head and neck surgeons, neurosurgeons, and plastic and reconstructive surgeons. The key to select an appropriate surgical procedure is dependent on whether the intracranial approach is needed or not. We demonstrated four types of surgical procedures for EAC cancer, as shown in Fig. 1 . Sleeve resection is a surgical technique that allows removal of the tumor with the surrounding tissue, and this technique is only performed for T1 tumors without bone invasion. LTBR is commonly used for T1 and T2 tumors, in which the cancer is resected with the surrounding external bone. Using LTBR, most T1 and T2 tumors were resected with acceptable survival results. 3 24 These procedures do not require the intracranial approach and are considered relatively safe. 24 Alternatively, a more aggressive surgical approach is needed for locally advanced cases, such as T3 or T4a tumors. Ex-LTBR was performed for locally advanced tumors with involvement of the mastoid bone or temporomandibular joint, in which the tumor was resected in an en bloc fashion by using both intra- and extracranial approaches. Despite the relatively longer operative time and intraoperative blood loss during surgery, Ex-LTBR allows removal of the tumor with the surrounding tissue, including the temporomandibular joint and the mastoid bone, to achieve tumor-free margins. Due to the complexity of the temporal bone, an en bloc resection with tumor-free margins is not easy for locally advanced-stage tumors such as T4 tumors. STBR is feasible and useful as a curative surgical method, especially for T4a tumors. Matoba et al also reported the effectiveness and safety of STBR, although STBR is a difficult surgical procedure and may lead to severe perioperative complications such as hemorrhages or meningitis. 12 Total temporal bone resection was previously performed, but has not been currently performed for locally advanced cases because of the risk of severe complications and the difficulty in performing an en bloc resection. 5 25 Considering the surgical impact of craniofacial resection in both the physical and psychological aspects of the patients with advanced-stage tumors, surgeons should carefully determine the surgical indication according to the patient's condition, age, and comorbidity in clinical settings.

Surgical margin status is thought to be an important prognostic factor in SCC of the EAC. 19 25 Although in this study, no prognostic impact for margin status was found between the 5-year OS and DSS ( *p* = 0.23 and 0.13), 4 (13%) of the 30 surgical patients showed close or positive margins on pathological assessment of resected tumor specimens. To achieve complete en bloc resection of the tumor, accurate preoperative evaluation of tumor invasion based on CT and MRI and careful discussion among a multidisciplinary skull base surgery team are essential. A recent surgical virtual simulation using patient's original CT images is very helpful for bone resection and tissue reconstruction in extended craniofacial resection in the head and neck cancer, 17 26 enabling surgeons to understand the complicated skull base anatomy and to teach the surgical technique to young surgeons.

In our study, 20% of patients received adjuvant radiation or chemoradiation. Previous studies have shown that aggressive primary surgical treatment with postoperative radiotherapy offers the greatest chance of cure for SCC of the temporal bone. 27 However, Lobo et al showed that no difference in survival outcome was found between the group treated with surgery plus adjuvant radiotherapy and the group treated only with surgery in stages III and IV. 28 Currently, it remains controversial whether postoperative radiotherapy is recommended for SCC of the EAC. 29 Most importantly, it is essential to determine whether complete tumor resection with free tumor margins is achieved on the resected tumor specimen after surgery. Therefore, pathologists and head and neck surgeons should closely discuss the patient's history and tumor invasion pattern and point out the location closest to the tumor after surgery. Postoperative radiotherapy should be considered an adjuvant therapy when a safe tumor margin is not obtained.

Extended surgical resection is highly invasive for patients because of the high amount of blood loss and the long operation time, which often results in prolonged complications including hearing loss, facial paralysis, and cosmetic deformities. The indirect negative effect of psychiatric disorders, such as depression, on the outcome of head and neck cancer treatment is also well known. 30 In particular, for malignant skull base tumors, early psychiatric intervention is essential to improve the health-related quality of life at preoperative and follow-up periods. 31 A multidisciplinary approach with a skull base surgery team and a liaison consultation team is essential for locally advanced EAC cancer patients. Early psychiatric interventions can be life-saving during the pre- and postoperative periods.

The main limitation of this study was that a relatively small number of EAC cancer patients were selected from a single center based on the retrospective results. Therefore, the results need to be confirmed in a larger sample size, selected from multiple centers.

## Conclusion

The present study focused on patients with SCC of the EAC at a university hospital over a period of 20 years, especially with skull base involvement, and a T4 subclassification was proposed. Complete tumor resection in an en bloc fashion could help achieve a good survival rate even in patients with locally advanced tumors.
