# Supplementary material for: Surgical Strategy for Squamous Cell Carcinoma of the External Auditory Canal: Management of Locally Advanced Cases with Skull Base Involvement
Source: J Neurol Surg B Skull Base. 2022 Feb 4;84(1):69–78. doi: 10.1055/a-1733-2585 (PMC9897898; doi:10.1055/a-1733-2585)
Supplement: Supplementary file 1 — Supplementary Material [file 10-1055-a-1733-2585-s210191.pdf]

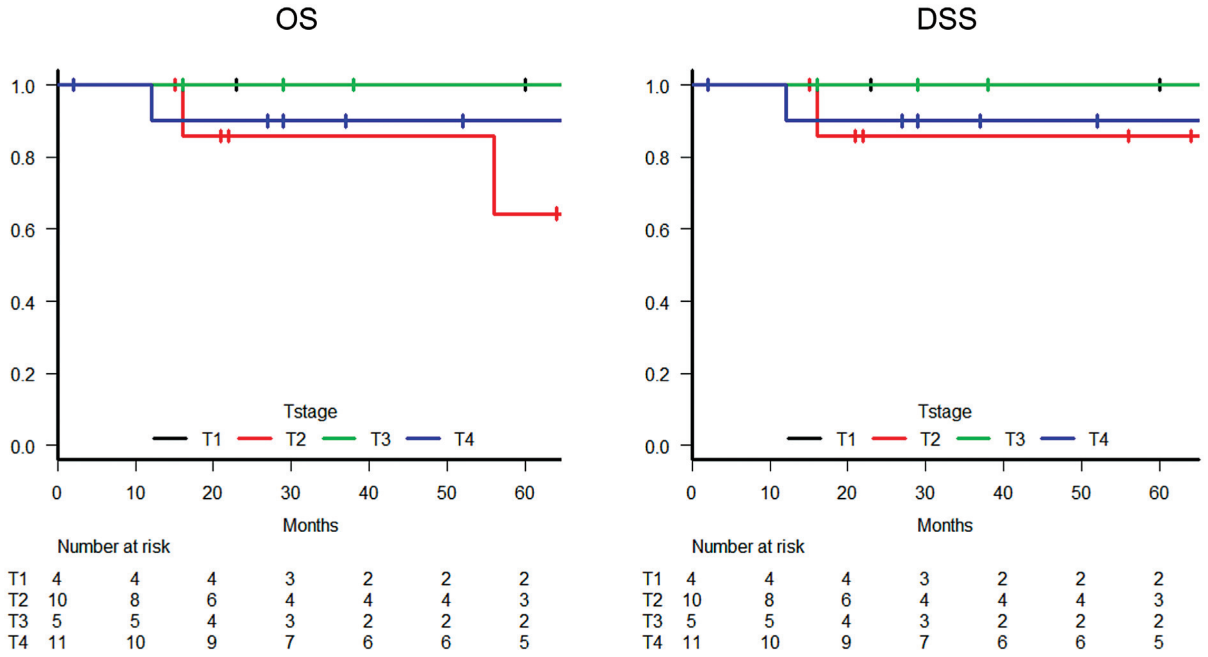

Supplemental Fig. S1 Kaplan–Meier survival curves based on the modified Pittsburgh classification for overall survival and disease-specific survival in 30 surgical patients with squamous cell carcinoma of the external auditory canal (DSS, disease-specific survival; OS, overall survival.)

Supplemental Table S1 Tumor invasion features in patients with T4 tumors who received surgery and those who did not

		Surgical T4 patients	Non-surgical T4 patients
		n = 11	*n* = 12
Anterior	Temporomandibular joint	8	11
	Parotid gland	4	10
	Styloid process	2	10
Medial	Medial wall of the middle ear	8	7
	Cochlea	0	2
	Petrous apex	0	4
	Carotid canal	0	5
	Jugular foramen	0	7
Posterior	Mastoid	3	11
	Facial nerve	2	6
	Sigmoid sinus	0	5
Upper	Dura	2	9
	Brain	0	1

 © 2022. The Author(s).
